# Graphene-Oxide-Modified Metal–Organic Frameworks Embedded in Mixed-Matrix Membranes for Highly Efficient CO_2_/N_2_ Separation

**DOI:** 10.3390/nano14010024

**Published:** 2023-12-21

**Authors:** Long Feng, Qiuning Zhang, Jianwen Su, Bing Ma, Yinji Wan, Ruiqin Zhong, Ruqiang Zou

**Affiliations:** 1 State Key Laboratory of Heavy Oil Processing, China University of Petroleum, No. 18 Fuxue Road, Changping District, Beijing 102249, China17860610076@163.com (J.S.);; 2 Beijing Key Laboratory for Theory and Technology of Advanced Battery Materials, School of Materials Science and Engineering, Peking University, No. 5 Yiheyuan Road, Haidian District, Beijing 100871, China

**Keywords:** mixed-matrix membranes, Pebax^®^1657 (polyether block amide), metal–organic framework, CO_2_/N_2_ separation

## Abstract

MOF-74 (metal–organic framework) is utilized as a filler in mixed-matrix membranes (MMMs) to improve gas selectivity due to its unique one-dimensional hexagonal channels and high-density open metal sites (OMSs), which exhibit a strong affinity for CO_2_ molecules. Reducing the agglomeration of nanoparticles and improving the compatibility with the matrix can effectively avoid the existence of non-selective voids to improve the gas separation efficiency. We propose a novel, layer-by-layer modification strategy for MOF-74 with graphene oxide. Two-dimensional graphene oxide nanosheets as a supporting skeleton creatively improve the dispersion uniformity of MOFs in MMMs, enhance their interfacial compatibility, and thus optimize the selective gas permeability. Additionally, they extended the gas diffusion paths, thereby augmenting the dissolution selectivity. Compared with doping with a single component, the use of a GO skeleton to disperse MOF-74 into Pebax^®^1657 (Polyether Block Amide) achieved a significant improvement in terms of the gas separation effect. The CO_2_/N_2_ selectivity of Pebax^®^1657-MOF-74 (Ni)@GO membrane with a filler concentration of 10 wt% was 76.96, 197.2% higher than the pristine commercial membrane Pebax^®^1657. Our results highlight an effective way to improve the selective gas separation performance of MMMs by functionalizing the MOF supported by layered GO. As an efficient strategy for developing porous MOF-based gas separation membranes, this method holds particular promise for manufacturing advanced carbon dioxide separation membranes and also concentrates on improving CO_2_ capture with new membrane technologies, a key step in reducing greenhouse gas emissions through carbon capture and storage.

## 1. Introduction

In recent years, the significant rise in global greenhouse gas emissions caused by industrial development has intensified environmental concerns and exacerbated climate change [1,2]. Accelerating the development of efficient and environmentally friendly gas separation technology is crucial for achieving carbon neutrality and supporting carbon capture, utilization, and storage (CCUS) efforts [3,4]. Membrane separation technology is particularly attractive due to its energy efficiency, environmental friendliness, and cost-effectiveness [5,6,7,8]. Mixed-matrix membranes, which are composites of organic polymers and inorganic particles, integrate the easy processability of polymer membranes with the enhanced permeability and selectivity of inorganic membranes. Nevertheless, homogeneous polymeric membranes are constrained by the intrinsic permeability–selectivity trade-off dilemma [9]. This hybrid structure has demonstrated potential for improved performance in gas separation applications [10].

Among the fillers, metal–organic frameworks (MOFs), which are a novel class of coordination polymers, have emerged as compelling fillers for the development of high-performance MMMs. This attraction stems from their exceptional attributes, including high porosity, tunable uniform micro-pores, mechanical stability, and chemical stability [11,12]. MOF-based MMMs are primarily used to distinguish gaseous molecules with similar molecular dimensions and/or physicochemical characteristics, such as O_2_, N_2_, CO_2_, and CH_4_. The integration of MOFs within these matrices is known to enhance selectivity and permeability, thereby facilitating the efficiency and efficacy of gas separation [13,14,15]. However, the interfacial affinity between the polymer and MOF particles decreases as the MOF content in the matrix increases. Concurrently, there is an increased tendency towards particle agglomeration, which consequently impairs the uniformity of the filler distribution [16,17]. To address this issue, a spectrum of surface modification techniques has been explored and implemented. The prevalent approach involves functionalizing MOFs, a process that not only enhances the separation selectivity of the MMMs, but also ameliorates the dispersion of MOFs within the membrane matrix [18,19,20]. However, functional groups that induce excessive hydrophilicity or hydrophobicity may lead to poor interfacial compatibility and phase separation [21].

Beyond functionalizing with organic groups, an alternative approach involves conjugating graphene oxide (GO) with metal–organic frameworks (MOFs). This method enhances the dispersion of MOFs and improves their interfacial adhesion within polymeric matrices [22,23,24]. GO is known to have a high aspect ratio, which can increase the path length, twists, and turns of the channels in MMMs, allowing only small molecules to pass and restricting larger ones [25]. Most notably, this method effectively addresses the common issues of inadequate polymer–filler compatibility and filler aggregation. Anastasiou et al. [25] developed PSF-based MMMs with a ZIF-8/GO filler using solution casting. This approach resulted in improving CO_2_ selectivity and permeability, specifically for CO_2_/N_2_ and CO_2_/CH_4_ gas separation. The microporosity of ZIF-8 promoted CO_2_ adsorption, while GO created a convoluted pathway that selectively permitted smaller CO_2_ molecules and hindered larger N_2_ and CH_4_ molecules, thereby enhancing gas separation efficiency. However, research on the gas separation performance of MOF@GO-based MMMs still has a long way to go.

In this study, we propose a strategy for modifying MOF-74 (Ni) with GO to bolster both gas diffusion and solubility selectivity of the MMMs. The MOF-74(Ni) grew in situ on the stratified platform of GO. And after uniform dispersion into the matrix membrane synthesized with Pebax^®^1657, the MMMs showed an excellent gas separation efficiency of 76.96 of CO_2_ towards N_2_. Different loadings of GO and MOF-74(Ni) on the separation efficiency of MMMs were systematically investigated, unveiling the trade-off between facilitated transport and separation selectivity. Furthermore, this circumvented the high energy demands and expensed associated with traditional separation technologies.

## 2. Experimental Methods

### 2.1. Materials

2,5-Dihydroxyterephthalic acid (H_4_DOBDC, 98%) was obtained from Energy Chemical (ZeSheng, Guangzhou, China). Nickel(II) nitrate hexahydrate (Ni(CH_3_COO)_2_·4H_2_O, AR) was obtained from Sinopharm Chemical Reagent Co., Ltd. (Sinopharm, Shanghai, China). GO aqueous dispersion (2 mg/mL) was purchased from XFNANO Nanotechnology Materials (Jiangsu) Co., Ltd. (XFNANO, Nanjing, China). Pebax^®^1657 was obtained from Arkema (Shanghai, China) Co., Ltd. (Arkema, Shanghai, China) and ethanol (AR) was supplied by TongGuang Fine Chemical Company (Beijing, China).

### 2.2. Synthesis of MOF-74(Ni)

**Scheme 1:**
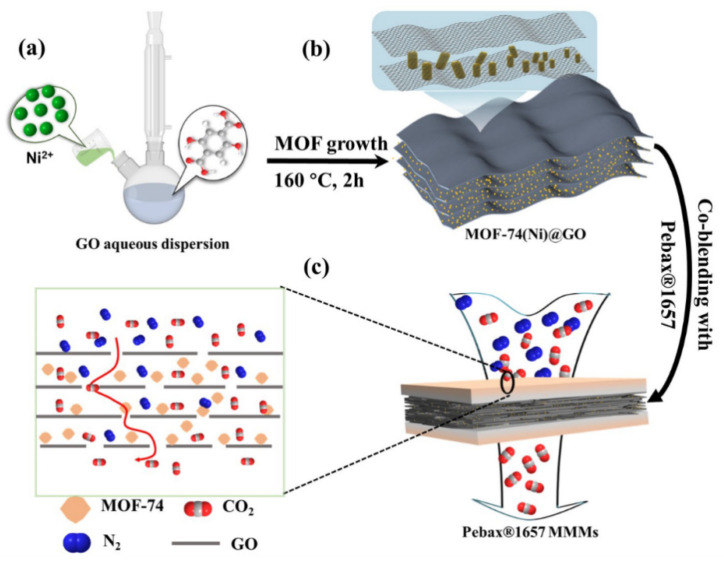
Synthesis processes of Pebax^®^1657-MOF-74(Ni)@GO. (**a**) MOF-74(Ni) grows on GO, (**b**) the structure of MOF-74(Ni)@GO, (**c**) the structure of MMMs.

MOF-74(Ni) was prepared through a one-pot method, as shown in Scheme 1a. First, H_4_DOBDC (5.16 g, 26 mmol) was dissolved in 200 mL of de-ionized water and then kept under reflux with constant stirring at 160 °C (heating device). In another flask, Ni(CH_3_COO)_2_·4H_2_O (12.60 g, 50 mmol) was dissolved in 50 mL of deionized water and heated to 80 °C with stirring. The Ni(CH_3_COO)_2_·4H_2_O was then transferred into the H_4_DOBDC solution. After refluxing for 2 h, the mixture was cooled to room temperature and washed three times with de-ionized water. The resulting yellow-colored MOF-74(Ni) nanoparticles were obtained by centrifugation, washed with ethanol, and dried under vacuum at 80 °C for 12 h.

### 2.3. Synthesis of MOF-74(Ni)@GO

The schematic illustration of the preparation of MOF-74@GO is shown in Scheme 1. Initially, H_4_DOBDC (0.516 g, 2.6 mmol) was dissolved in a 17.60 mL GO aqueous dispersion, followed by ultrasonic mixing for 10 min. This mixture was then refluxed at 160 °C (heating device) for 2 h. Concurrently, a solution of Ni(CH_3_COO)_2_·4H_2_O (12.60 g) in 5 mL deionized water was introduced into the flask at 80 °C, and the mixture was further refluxed for an additional 2 h. The resultant precipitate was centrifuged and repeatedly washed with deionized water until a neutral pH was achieved. A dark green powder, designated as MOF-74(Ni)@GO-1, was obtained through centrifugation, washed with ethanol, and freeze-dried.

MOF-74(Ni)@GO composites were synthesized with varying GO loadings, achieved by adjusting the volume of GO aqueous dispersion to 17.60 mL, 35.20 mL, and 53.28 mL. These were designated as MOF-74(Ni)@GO-1, MOF-74(Ni)@GO-2, and MOF-74(Ni)@GO-3, respectively. 

### 2.4. Fabrication of Composite MMMs

#### 2.4.1. Preparation of Pebax^®^1657 Polymer Membrane

The Pebax^®^1657 polymer membrane was fabricated using a solvent casting method. Pebax^®^1657 polymer (5 g) was dissolved in an ethanol (88.7 mL) and deionized water (30 mL) mixture, and then heated to 80 °C with continuous stirring under reflux for 3 h. The resulting 5 wt% Pebax^®^1657 solution, termed the casting solution, was then applied to a polytetrafluoroethylene (PTFE) mold. After solvent evaporation, the membrane was gently removed from the mold. A final drying step at 40 °C for 12 h produced a Pebax^®^1657 polymer membrane with a thickness of 70 μm.

#### 2.4.2. Preparation of MMMs

MMMs were produced using blending and solution casting techniques, following the method outlined for Pebax^®^1657 polymer membrane fabrication (see Scheme 1). For these membranes, varying quantities of fillers were incorporated into a 5 wt% Pebax^®^1657 casting solution, accompanied by 30 min of ultrasonic dispersion. The following steps were mirrored for Pebax^®^1657 membrane production.

Different MMMs were synthesized by adding specific amounts of MOF-74(Ni) (0.0264 g, 0.0559 g, 0.0890 g, and 0.1260 g), resulting in varying MOF loadings. These were designated as Pebax^®^1657-MOF-74(Ni)-x%, where x indicated the MOF loading percentage (5, 10, 15, or 20). Similarly, substituting MOF-74(Ni) with MOF-74(Ni)@GO composites led to the successful preparation of Pebax^®^1657-MOF-74(Ni)@GO membranes.

## 3. Results and Discussion

### 3.1. Characterization of MOF-74(Ni) and MOF-74(Ni)@GO

**Figure 1:**
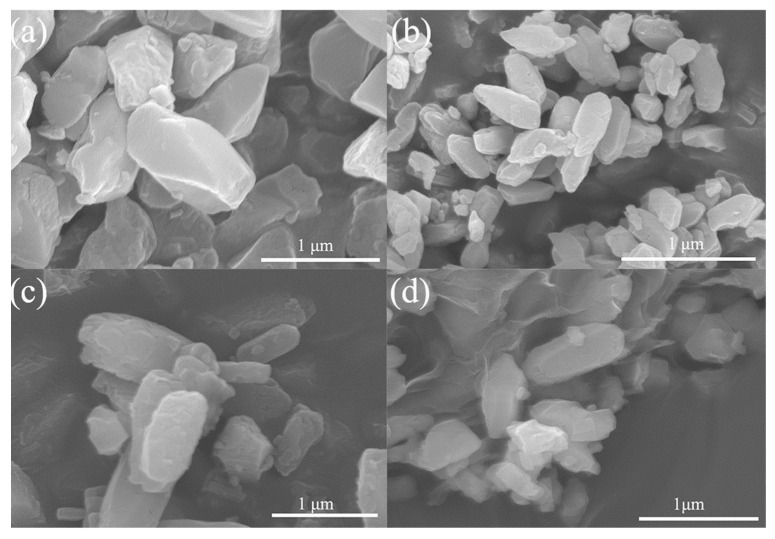
SEM images of (**a**) MOF-74(Ni), (**b**) MOF-74(Ni)@GO-1, (**c**) MOF-74(Ni)@GO-2, and (**d**) MOF-74(Ni)@GO-3.

The SEM images in Figure 1 revealed the nanorod structure of MOF-74(Ni) and its composites with varying GO loadings. The images of MOF-74@GO revealed distinct wrinkles and folds on the surface. This change was attributed to the strong interaction between the metal sites of MOFs and the epoxy groups on GO. These epoxy groups played a dual role in MOF-74(Ni) crystallization: firstly, as nucleation sites for crystal growth initiation, and secondly, as promoters of crystal growth and development [26]. This interaction inhibited crystallite aggregation and enhanced dispersion, leading to smaller, well-distributed MOF-74(Ni) crystallites. As demonstrated in Figure 1 and Table S1, MOF-74(Ni)@GO-2 exhibited the smallest crystal size and the highest surface area among the composites. In contrast, MOF-74(Ni)@GO-3 had a similar size, but the lowest surface area, with a significant presence of layered GO. This observation suggested incomplete GO dispersion in the composite, causing structural distortions. The addition of GO improved MOF dispersion and interfacial compatibility with the polymer matrix, thereby effectively reducing MOF particle agglomeration.

**Figure 2:**
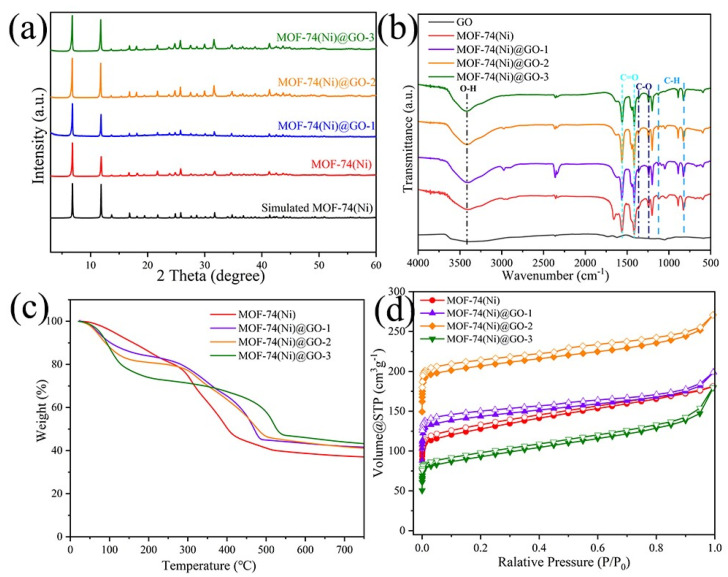
The (**a**) XRD, (**b**) FTIR, (**c**) TG, and (**d**) N_2_ adsorption and desorption isotherms at 77 K of MOF-74(Ni) and the series of MOF-74(Ni)@GO.

The structure of MOF particles were verified by X-ray diffraction (XRD), as shown in Figure 2a. MOF-74(Ni) exhibited two characteristic peaks at 6.8° and 11.9°, aligning with both simulated MOF-74(Ni) patterns and previous reports [27,28]. These sharp, well-defined peaks indicated the high crystallinity of MOF-74(Ni). Notably, the peak positions and intensities remained unchanged after GO modification, confirming the preservation of the original structure and illustrating the crystalline maintenance. The crystal structure of MOF-74 (Ni)@GO remained intact when integrated into a Pebax polymer matrix. In addition, the prepared MMMs had good stability and tensile strength (Figure S3). The Ni content in the composite materials was accurately quantified using inductively coupled plasma mass spectrometry (ICP-MS), facilitating the determination of MOF-74 and GO proportions in the composites. Consequently, the calculated percentages of GO in MOF-74@GO-1, MOF-74@GO-2, and MOF-74@GO-3 were found to be 0.83 wt%, 1.30 wt%, and 1.72 wt%, respectively.

Fourier transform infrared spectroscopy (FTIR) analysis, as shown in Figure 2b, further confirmed the interaction between GO and MOF-74(Ni) during the reaction process. The peak at 3400 cm^−1^ was attributed to the presence of coordination water and crystal water within the materials [29]. The peaks observed at 1560 cm^−1^ and 1415 cm^−1^ were associated with the asymmetric and symmetric stretching vibrations of C=O in the coordinated carboxyl group, while the asymmetric and symmetric stretching vibrations of C-O were evident at 1363 cm^−1^ and 1242 cm^−1^ [30,31]. Additionally, the peak at 826 cm^−1^ originated from the out-of-plane bending vibration of C–H on benzene rings [32]. After the GO coating, the characteristic peak of MOF-74(Ni) remained discernible in the spectra, and no new peaks emerged. The spectra around 3390 cm^−1^ and 1735 cm^−1^ were assigned to the O-H stretching vibration and C=O stretching vibration of the carboxyl group in the GO, respectively. Notably, in the MOF-74(Ni)@GO composite, as the GO content increased, the peak intensity at 1735 cm^−1^ diminished. This reduction suggested an interaction between the carboxyl groups of GO and Ni 2p, providing further affirmation of the successful growth of MOF-74(Ni) on the GO substrate.

The thermogravimetric analysis curves (TGA) in Figure 2c illustrated the thermal stability of both MOF-74(Ni) and its composites, showing a two-step weight loss process. The first significant weight loss, accounting for about 10% of the total, occurred below 150 °C. This loss was primarily due to the desorption of water and solvent molecules from the pores [33]. This desorption extended from 150 °C to 280 °C, and was associated with molecules binding to the unsaturated Ni^2+^ sites [34]. A second major weight loss, comprising approximately 35% of the total, was observed between 280 °C and 400 °C, attributed to the breakdown of the crystal framework [35]. The range of 400 °C to 525 °C saw further degradation of the crystal structure. After calcination at 700 °C in O_2_, the final residue (30.30%) of MOF-74(Ni) was identified as NiO. In the case of MOF-74(Ni)@GO, the residue contents were 30.06%, 29.96%, and 29.85%, and GO had been burned off. The actual percentages of GO in MOF-74(Ni)@GO were calculated to be 0.79 wt%, 1.12 wt%, and 1.49 wt% (detailed calculations were shown in Equations (S5) and (S6)), which were very close to the ICP test results.

To assess the effect of GO loading on the specific surface area, pore volume, and pore distribution of the composite materials, the MOF-74(Ni) and composites were further characterized through nitrogen adsorption–desorption analysis, as illustrated by Figure 2d and summarized in Table S1. As shown in Figure 2d, all the samples demonstrated a pronounced adsorption capacity at an extremely low *P*/*P*_0_ (<0.05), characteristic of a type I adsorption equilibrium isotherm, as per the IUPAC classification. This suggested that the materials were all microporous. As shown in Table S1, the N_2_ adsorption capacity of MOF-74(Ni)@GO-1&2 was higher compared to MOF-74(Ni), signifying an increase in porosity following the incorporation of GO into the composites. Specifically, MOF-74(Ni)@GO-2 displayed a surface area of 837.34 m^2^g^−1^ and a pore volume of 0.42 cm^3^g^−1^, showing remarkable increases of 73.69% and 49.57%, respectively, compared to the original material. This increase was attributed to improved pore accessibility due to the presence of missing cluster defects. Conversely, higher GO loadings resulted in reduced surface areas; MOF-74(Ni)@GO-3, with the highest GO content, had the lowest surface area of 343.99 m^2^g^−1^, likely due to GO agglomeration [36]. As indicated by the data presented in Table S1, the fluctuations in micropore volume aligned with changes in total pore volume. Additionally, the introduction of GO (as depicted in Figure S1) was associated with an increase in the pore width, indicating a potential enhancement of pore formation facilitated by GO.

### 3.2. Characterization of MMMs

Figure S2 and Table S2 demonstrated that the pristine Pebax^®^1657 membrane was transparent; flexible; and featured a smooth, nonporous surface, making it an ideal matrix material for membrane separation. Compared to the original Pebax^®^1657 membrane, the MMMs showed changes in color that aligned with the respective fillers, as depicted in Figure S3. Importantly, there was no visible agglomeration of filler on the membrane surface, suggesting effective dispersion of the filler within the Pebax^®^1657 matrix.

Figure S4 presented SEM images that detail the morphology of MMMs, highlighting the effects of various fillers and their loadings. With MOF-74(Ni) as a filler, the particles were dispersed across the polymer matrix surface, but also showed signs of agglomeration, negatively impacting gas permeation. Conversely, at a 10 wt% MOF-74(Ni)@GO loading, the morphology was uniform and continuous, with no evidence of agglomeration or defects. This uniformity suggested an effective combination of polymer and filler, implying that GO positively influenced the interaction between them. This interaction significantly reduced aggregation and defects, thereby enhancing the integration of the filler with the polymer.

**Figure 3:**
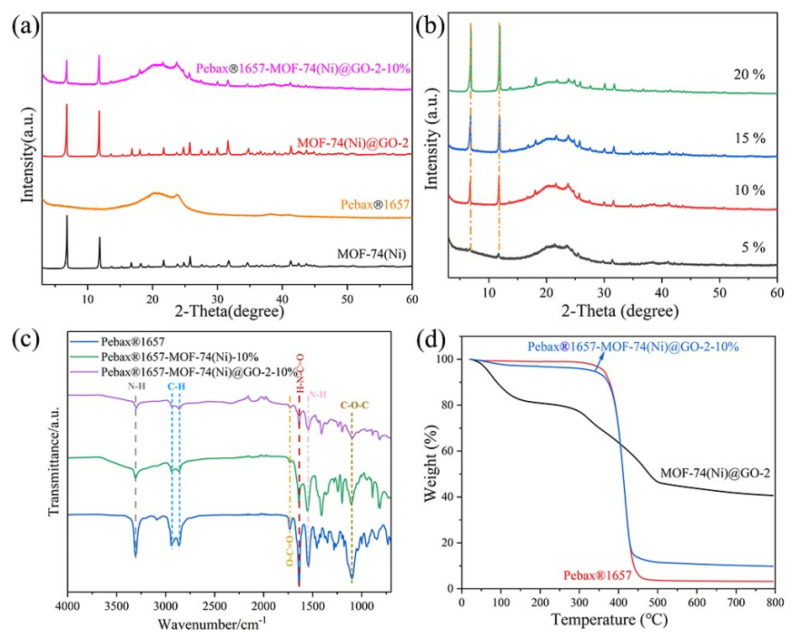
XRD patterns of (**a**) MMMs with different fillers, (**b**) composite membranes with different loadings of MOF-74(Ni)@GO-2, (**c**) FTIR patterns, and (**d**) TGA curves of MOFs and membrane samples.

Figure 3 illustrated the chemical compatibility analysis of each component in the composite matrix membrane using XRD. The diffraction peaks of MOF-74(Ni) and MOF-74(Ni)@GO-2 were sharp and intense, indicative of their highly crystalline nature. In contrast, the Pebax^®^1657 membrane showed a broad peak between 15° and 25°, confirming its amorphous character [37,38]. The characteristic peak of MOF-74(Ni) remained visible in the XRD pattern of the Pebax^®^1657-MOF-74(Ni)-10% membrane, indicating that MOF-74(Ni) preserved its crystallinity post-membrane fabrication. Similarly, the MOF-74(Ni)@GO series maintained its integrity during the membrane’s preparation, as seen in Figure 3. Additionally, Figure 3b and Figure S5 revealed an increase in the intensity of the characteristic diffraction peaks as the filler loading rose from 5% to 20%, suggesting the physical mixing of the composite material.

FTIR analysis, as depicted in Figure 3c, was performed to examine the composition and physical binding of the composite matrix membrane. For pristine Pebax^®^1657, the band at 1099 cm^−1^ was due to the stretching vibration of C-O-C groups in PEO. Peaks at 1544 and 1638 cm^−1^ were associated with the -NH in PA, and the peak at 1733 cm^−1^ corresponded to the C=O bond from the rigid PA and the ester. Symmetric and asymmetric stretching vibration peaks of the C-H bonds in aliphatic chains were observed at 1943 and 1864 cm^−1^, respectively. Additionally, N-H stretching vibration peaks were observed at 3308 cm^−1^ [39,40,41]. The characteristic peaks of Pebax^®^1657-MOF-74(Ni)-10% and Pebax^®^1657-MOF-74(Ni)@GO-10% aligned with those of pristine Pebax^®^1657 and MOF-74(Ni), showing no new peaks or shifts. This alignment suggested that the interactions between Pebax^®^1657 and the fillers were purely physical, corroborating the XRD findings and confirming the successful creation of the MMMs.

Figure 3d showcased the thermal stability of Pebax^®^1657, revealing a two-step thermal degradation process. The initial weight loss was attributed to the volatilization of water and ethanol. The primary degradation step, occurring between 330 and 430 °C, corresponded to the breakdown of the soft PEO segment. Above 430 °C, a minor weight loss in the hard PA segment was observed, underscoring its contribution to the mechanical and thermal stability of the polymer membranes [42,43]. As depicted in Figure S6, membranes with various fillers and filler loadings exhibited similar behavior to neat Pebax^®^1657, all demonstrating a consistent one-step decomposition pattern. These TGA results suggested that MOF incorporation slightly alters the thermal properties, which was advantageous for preserving the gas permeability of the blended membranes.

**Figure 4:**
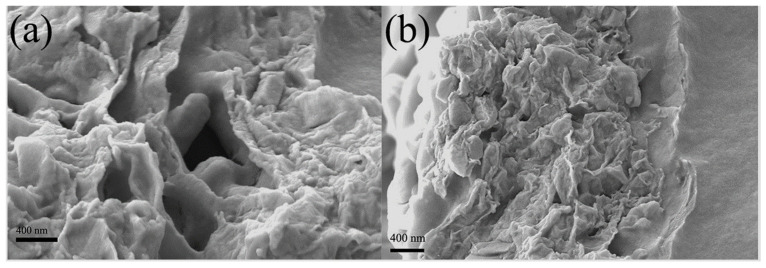
The cross-sectional morphology of (**a**) neat Pebax^®^1657 and (**b**) Pebax^®^1657-MOF-74(Ni)@GO-2-10%.

Figure 4a revealed that the pristine Pebax membrane had a porous microstructure, characterized by an irregular network of voids and channels. In contrast, as shown in the MMMs, there was a densely packed aggregation of particulates with closely spaced inter-particulate boundaries. Despite this dense packing, the filler particles within the MMMs were uniformly dispersed, retaining their original shape and size. This structural configuration, with minimized voids, was beneficial for enhancing selective gas separation.

### 3.3. Characterization of Gas Separation

**Figure 5:**
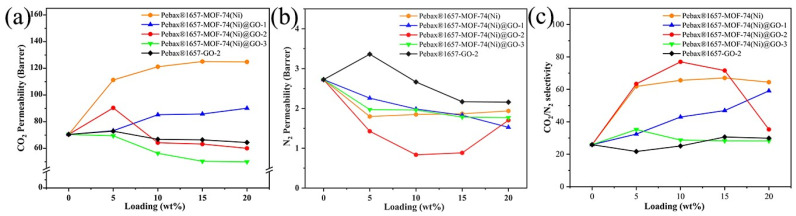
(**a**) CO_2_, (**b**) N_2_ permeability, and (**c**) CO_2_/N_2_ selectivity of MMMs with different fillers and filler loadings (the number of replicates for each data point is three).

The pure gas permeation experiments were performed at 25 °C and 2 bar using the permeation rig schematic (Scheme S1) to assess CO_2_, N_2_ permeability, and CO_2_/N_2_ selectivity among MMMs with different fillers and filler loadings. The results can be found in in Figure 5 and Tables S3–S5. It can be seen that the introduction of MOF-74(Ni)@GO significantly influenced the transport properties of the membranes. In Figure 5, as the filler content in the membranes increased, there was a gradual increase in the permeability of CO_2_ while the permeability of N_2_ decreased. The enhancement of CO_2_ permeability can be attributed to several factors. Firstly, the high porosity and flexible framework of MOF-74(Ni) facilitated the diffusion of CO_2_ molecules, thereby enhancing the overall CO_2_ permeability. In addition, the presence of polar functional groups on GO enabled specific interactions with CO_2_, promoting its transport through the membrane. Finally, the incorporation of MOF-74(Ni)@GO introduced more intricate transmission pathways within the membrane, thus hindering the transport of a small fraction of CO_2_. However, this hindrance proved to be fatal for larger-sized N_2_ molecules [44,45,46]. Meanwhile, the first two factors selectively enhanced the permeability of CO_2_, and their effects were significantly stronger compared to the negative impact caused by the introduction of more complex pathways. As a result, a more pronounced increase in CO_2_ permeability, rather than a decrease, was observed in comparison to N_2_. The CO_2_ permeability of Pebax^®^1657-MOF-74(Ni)@GO-3 was lower than that of the neat Pebax^®^1657 membrane due to the increase in tortuosity caused by the incorporation of MOF-74(Ni)@GO-3. This resulted in the deterioration of gas diffusivity and subsequently led to reduced gas permeability [47]. The CO_2_ permeability of the Pebax^®^1657-MOF-74(Ni)@GO-3 composite was found to be lower compared to the pristine Pebax^®^1657 membrane. This decrease in permeability was attributed to the introduction of MOF-74(Ni)@GO-3, which increased the tortuosity within the material. The increased tortuosity had a stronger effect than the specific selectivity towards CO_2_, resulting in worsening gas diffusivity and consequently leading to lower gas permeability.

When comparing all the mixed-matrix membranes (MMMs), the Pebax^®^1657-MOF-74(Ni)@GO-2 composite with a 10% loading exhibited the highest CO_2_/N_2_ selectivity among the membranes which were investigated. In comparison to the neat Pebax^®^1657 membrane, the CO_2_/N_2_ selectivity increased by 197.26%. However, it is important to note that, at higher MOF-74(Ni)@GO-2 contents, such as those exceeding 15%, a significant number of aggregations or defects would be formed. These aggregations or defects could deteriorate the selectivity of the MMMs, as illustrated in Figure S4k,l,o,p.

To understand the enhanced selectivity which we observed, the solubility and diffusivity coefficients of Pebax^®^1657 and MMMs containing 10 wt% filler were examined according to Equations (S3) and (S4) [45]. The addition of fillers, especially to Pebax^®^1657-MOF-74(Ni)@GO-2-10%, significantly influenced the selectivity as shown in Table 1. The improvement in CO_2_/N_2_ selectivity was attributed to two key factors: increased solubility and reduced diffusion. The nanopore size of MOF-74(Ni)@GO-2 facilitated a size-sieving effect, restricting N_2_ penetration. Additionally, CO_2_ adsorption followed Langmuir adsorption, indicating that CO_2_ molecules predominantly underwent monomolecular surface diffusion through pore channels. These observations implied that gas separation in MMMs operated via a dissolution–diffusion mechanism. The inability to test this in a dusty environment means that the robustness and efficiency of the membranes under such conditions remain unverified.

**Figure 6:**
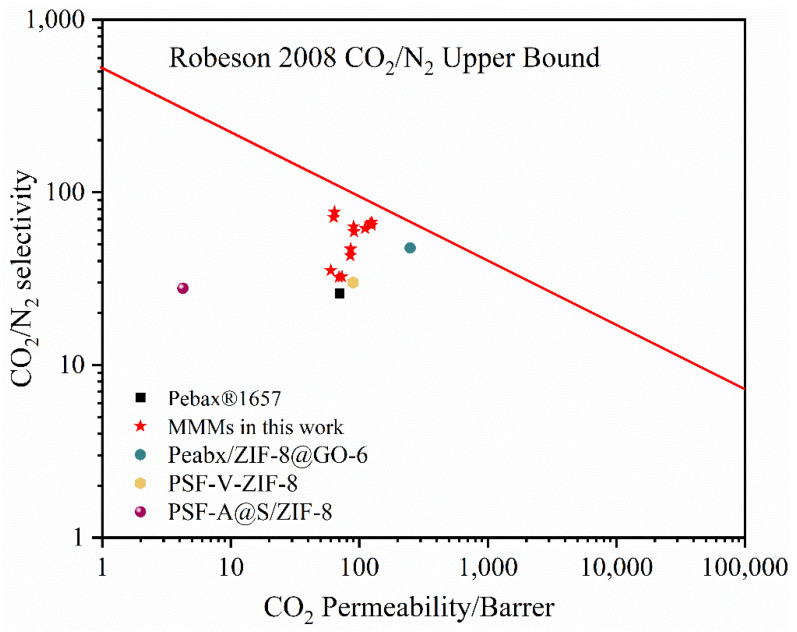
Comparison of CO_2_/N_2_ separation performance.

Figure 6 and Table S6 illustrate the separation performance plotted against the Robeson upper bound of 2008 [9]. Although the prepared MMMs showed significantly improved performance compared to Pebax^®^1657, the overall performance still did not surpass Robeson’s upper bound. Specifically, the Pebax^®^1657-MOF-74(Ni)@GO-2-10% membrane exhibited the best CO_2_/N_2_ separation performances, approaching the upper bound. The separation efficiency and sensitivity of the membrane when separating between different gases suggest that it could potentially be adapted for sensing applications.

## 4. Conclusions

In conclusion, we reported high-performance MMMs for the efficient separation of CO_2_/N_2_ by impregnating GO-modified MOF-74(Ni) into a Pebax^®^1657 matrix. The optimal inclusion of 10 wt% MOF-74(Ni)@GO-2 in the MMMs led to a substantial increase in CO_2_/N_2_ separation selectivity, achieving a 196.72% enhancement over the pristine Pebax^®^1657 membrane. This significant improvement was primarily due to the synergistic interplay between GO and MOF-74(Ni), which bolstered interfacial compatibility, curtailed nonselective defects, and deterred MOF agglomeration. Furthermore, the GO contributed to enhanced diffusivity selectivity, while the MOF-74(Ni), with its OMSs, amplified both the gas permeability and solubility selectivity of the membranes. Notably, a GO concentration exceeding 4 wt%, specifically at 6 wt%, inversely affected the separation selectivity, resulting in a 37.37% reduction. This finding underscores the critical need for precise GO concentration optimization in MMM fabrication. The successful incorporation of 4 wt% MOF-74(Ni)@GO into Pebax^®^1657 membranes not only exemplifies a robust strategy for augmenting gas separation performance, but also sheds light on the versatile applications of MOF-74(Ni)@GO composites in CO_2_ capture. The insights gleaned from this investigation are substantial, offering a feasible and scalable approach that could significantly improve the efficacy of gas separation processes within the industrial sector. This study paves the way for future research to explore the full potential of MOF-based composites in environmental and energy applications.

## Figures and Tables

**Table 1:** Solubility and diffusivity coefficients of Pebax^®^1657 and MMMs.

Sample	Solubility ×10^8^		Diffusivity ×10^−8^		α(CO_2_/N_2_)
	CO_2_	N_2_	CO_2_	N_2_	
Pebax^®^1657	7.45 ± 0.4	0.24 ± 0.2	9.47 ± 0.9	11.47 ± 1.3	25.89 ± 1.2
Pebax^®^1657-MOF-74(Ni)	9.99 ± 1.1	0.12 ± 0.1	12.23 ± 2.3	12.13 ± 1.4	65.58 ± 1.5
Pebax^®^1657-MOF-74(Ni) @GO-1-10%	12.89 ± 0.5	0.48 ± 0.2	6.61 ± 1.6	4.18 ± 0.8	42.94 ± 1.7
Pebax^®^1657-MOF-74(Ni) @GO-2-10%	8.06 ± 0.6	0.13 ± 0.1	7.98 ± 1.3	6.38 ± 1.4	76.96 ± 1.3
Pebax^®^1657-MOF-74(Ni) @GO-3-10%	7.87 ± 1.2	0.15 ± 0.1	7.07 ± 2.2	1.63 ± 0.4	28.76 ± 1.5

Diffusivity coefficient (cm^2^/s); solubility coefficient (cm^3^(STP) per cm^3^ per cm Hg).

## Data Availability

The data presented in this study are available upon request from the corresponding author.

## Supplementary Materials

The supporting information can be downloaded at: https://www.mdpi.com/article/10.3390/nano14010024/s1, Scheme S1: The schematic diagram of the permeation rig. Figure S1: The pore size distribution curves of MOF-74(Ni) and MOF-74(Ni)@GO with different GO loadings. Figure S2: (a) Apparent picture and (b) SEM pattern of Pebax®1657 membrane. Figure S3: Pebax®1657-MOF-74(Ni) membranes with different filler loadings (a–d); Pebax®1657-MOF-74(Ni)@GO-1 MMMs with different filler loadings (e–h); Pebax®1657-MOF-74(Ni)@GO-2 MMMs with different filler loadings (i–l); Pebax®1657-MOF-74(Ni)@GO-3 MMMs with different filler loadings (m–p). Figure S4: SEM images of Pebax®1657-MOF-74(Ni) membranes with different filler loadings (a–d); SEM images of Pebax®1657-MOF-74(Ni)@GO-1 MMMs with different filler loadings (e–h); SEM images of Pebax®1657-MOF-74(Ni)@GO-2 MMMs with different filler loadings (i–l); SEM images of Pebax®1657-MOF-74(Ni)@GO-3 MMMs with different filler loadings (m–p). Figure S5: XRD patterns of composite membrane materials (a) MOF-74(Ni), (b) MOF-74(Ni)@GO-1, (c) MOF-74(Ni)@GO-2, and (d) MOF-74(Ni)@GO-3. Figure S6: TGA curves of the membranes containing different (a) filler loading and (b) fillers. Table S1: BET surface area and pore volume of MOF-74(Ni) and MOF-74(Ni)@GO with different GO loadings. Table S2: The thickness of MMMs. Table S3: The permeability and ideal selectivity of gases for Pebax®1657 and mixed matrix membranes. Table S4: The permeability and ideal selectivity of gases for Pebax®1657-GO, which is the same as the GO loading in Pebax^®^1657-MOF-74(Ni)@GO-2. Table S5: The permeability and ideal selectivity of gases for MMMs with 10 wt% filler loadings. Table S6: Comparison of gas separation performance of this work with those of reported MMMs. References [47,48,49,50].
