# Supplementary material for: Graphene-Oxide-Modified Metal–Organic Frameworks Embedded in Mixed-Matrix Membranes for Highly Efficient CO2/N2 Separation
Source: Nanomaterials (Basel). 2023 Dec 21;14(1):24. doi: 10.3390/nano14010024 (PMC10780323; doi:10.3390/nano14010024)
Supplement: Supplementary file 1 [file nanomaterials-14-00024-s001.zip › nanomaterials-2753124-supplementary.pdf]

nanomaterials

MDPI

Supplementary Materials

Graphene-Oxide-Modified Metal–Organic Frameworks Embedded in Mixed-Matrix Membranes for Highly Efficient CO_2/N_2 Separation

Long Feng [1], Qiuning Zhang [1], Jianwen Su [1], Bing Ma [2], Yinji Wan [1], Ruiqin Zhong [1,*] and Ruqiang Zou [2,*]

[1] State Key Laboratory of Heavy Oil Processing, China University of Petroleum, Beijing, No. 18 Fuxue Road, Changping District, Beijing 102249, China; 17860610076@163.com (J.S.)
[2] Beijing Key Laboratory for Theory and Technology of Advanced Battery Materials, School of Materials Science and Engineering, Peking University, No. 5 Yiheyuan Road, Haidian District, Beijing 100871, China
* Correspondence: rzhong@cup.edu.cn (R.Z.); rzou@pku.edu.cn (R.Z.)

1. Material Characterizations

The micromorphology of MMMs was examined using a field-emission scanning electron microscope (FE-SEM, S-4800 and Regulus 8220, Hitachi, Tokyo, Japan).

Powder X-ray diffraction (XRD, Smart Lab, Rigaku, Japan) measurements with were carried out to analyze the structure of the prepared MMMs. The accelerating voltage and the applied currents were 45 kV and 200 mA, respectively. The initial scattering angle was from 3° to 60°, and the scanning speed was 10° min^{-1}.

For the Fourier Transform Infrared Spectroscopy (FTIR)analysis, we employed the Attenuated Total Reflectance (ATR) technique. The chemical structure of both the MOF-74(Ni)@GO power and Pebax®1657-MOF-74(Ni)@GO MMMs was analyzed using the Fourier transform infrared spectroscopy (INVENIOR, Platinum-ATR from Bruker co., Germany) within the wavenumber range of 4000 to 500 cm^{-1}.

Thermogravimetric analysis (TGA) was performed with SDT Q600 (TA, U.S.A), under N_2 atmosphere. The membrane samples were heated to 800°C, at a heating rate of 10°/min, with a N_2 flow of 100 mL/min.

Inductively Coupled Plasma Mass Spectrometry (ICP-MS, Prodigy 7, Leeman) was carried out to analyze the Ni contents in MOF-74(Ni)@GO to calculate the percentages of GO in MOF-74@GO.

N_2 adsorption-desorption isotherms were collected at 77 K on a Quantachrome Autosorb-IQ2 analyzer. Surface area, pore size distribution (PSD), and pore volume were calculated using the Brunauer-Emmett-Teller (BET) method, Density Functional Theory (DFT) and the t-plot method.

Pure gas permeability measurements were carried out for N_2 and CO_2 with a gas permeability tester (LM-V2, Lanmo Machinery Equipment Co., Ltd, Hunan). The gas permeability was calculated by the constant volume/variable pressure method at 25°C and 2 bar.

2. Gas Separation Experiments

Pure gas permeability measurements were carried out for N_2 and CO_2 with a gas permeability tester (LM-V2, Lanmo Machinery Equipment Co., Ltd, Hunan). The gas permeability was calculated by the constant volume/variable pressure method at 25°C and 2 bar. When the permeation reached a steady state, the permeability can be calculated as Eq. (1):

$$P = \frac{273\times10^{10}}{760} * \frac{VL}{AT\left(\frac{p_2 * 76}{14.7}\right)} * \frac{d_p}{d_t} \quad \text{(S1)}$$

Where P is the permeability coefficient (Barrer), V is the Downstream volume of the measurement (71.38 cm^{-3}), L is the thickness of the membrane (cm), A is the effective membrane area (cm^2), T is the temperature (K), P_2 is the intake pressure (Psia), $\frac{d_p}{d_t}$ is the change rate of pressure in the permeate compartments with time.

The ideal gas selectivity ($\alpha_{A/B}$) was defined as the pure gas permeability ratio of A to that of B, as Eq. (2):

$$\alpha_{A/B} = \frac{P_A}{P_B} \quad \text{(S2)}$$

The diffusivity and solubility of gas could be calculated by the solution-diffusion model, as Eq. (3) and Eq. (4):

$$D = \frac{L^2}{6\theta} \quad \text{(S3)}$$

$$S = \frac{P}{D} \quad \text{(S4)}$$

Where D is the gas diffusivity, L is the thickness of the membrane, and θ is the lag time. S is solubility, and P is the partial pressure of the gas.

3. Calculation of MOF-74(Ni) and GO Percentages in the Composites via TGA Results

The percentage of MOF-74(Ni) and GO in the composites was obtained by the following two equations.

$$\text{MOF\%} + \text{GO\%} = 1 \quad \text{(S5)}$$

$$\text{Residue}_{\text{MOF}}\% * \text{MOF\%} = \text{Residue\%} \quad \text{(S6)}$$

where MOF% and GO% were the weight percentages of MOF-74(Ni) and GO in the composites, respectively, and Residue% was the weight percentage of residue in composite.

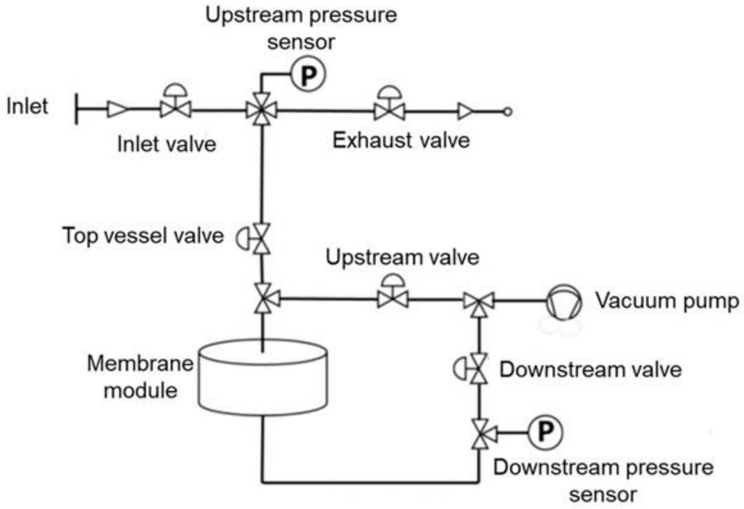

Scheme S1. The schematic diagram of the permeation rig.

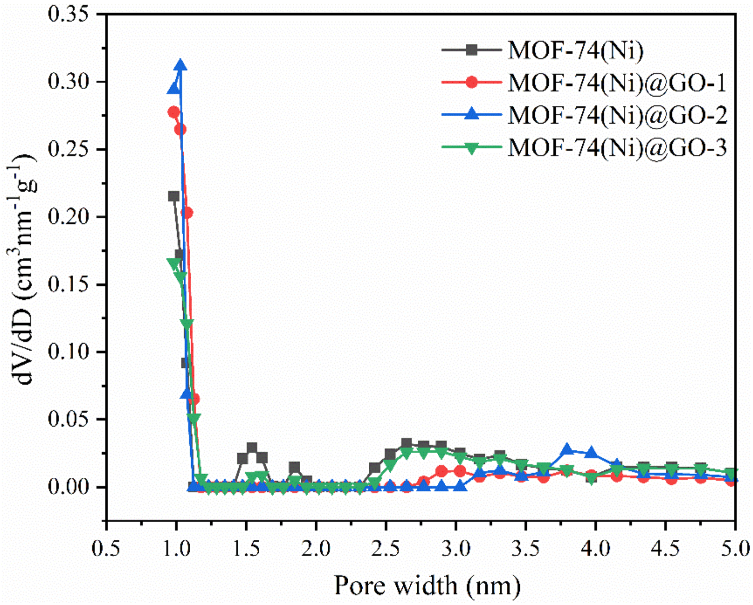

Figure S1. The pore size distribution curves of MOF-74(Ni) and MOF-74(Ni)@GO with different GO loadings.

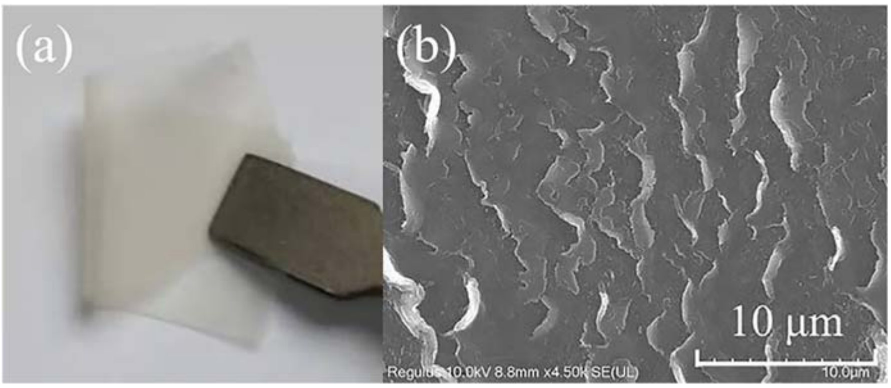

Figure S2. (**a**) Apparent picture and (**b**) SEM pattern of Pebax®1657 membrane.

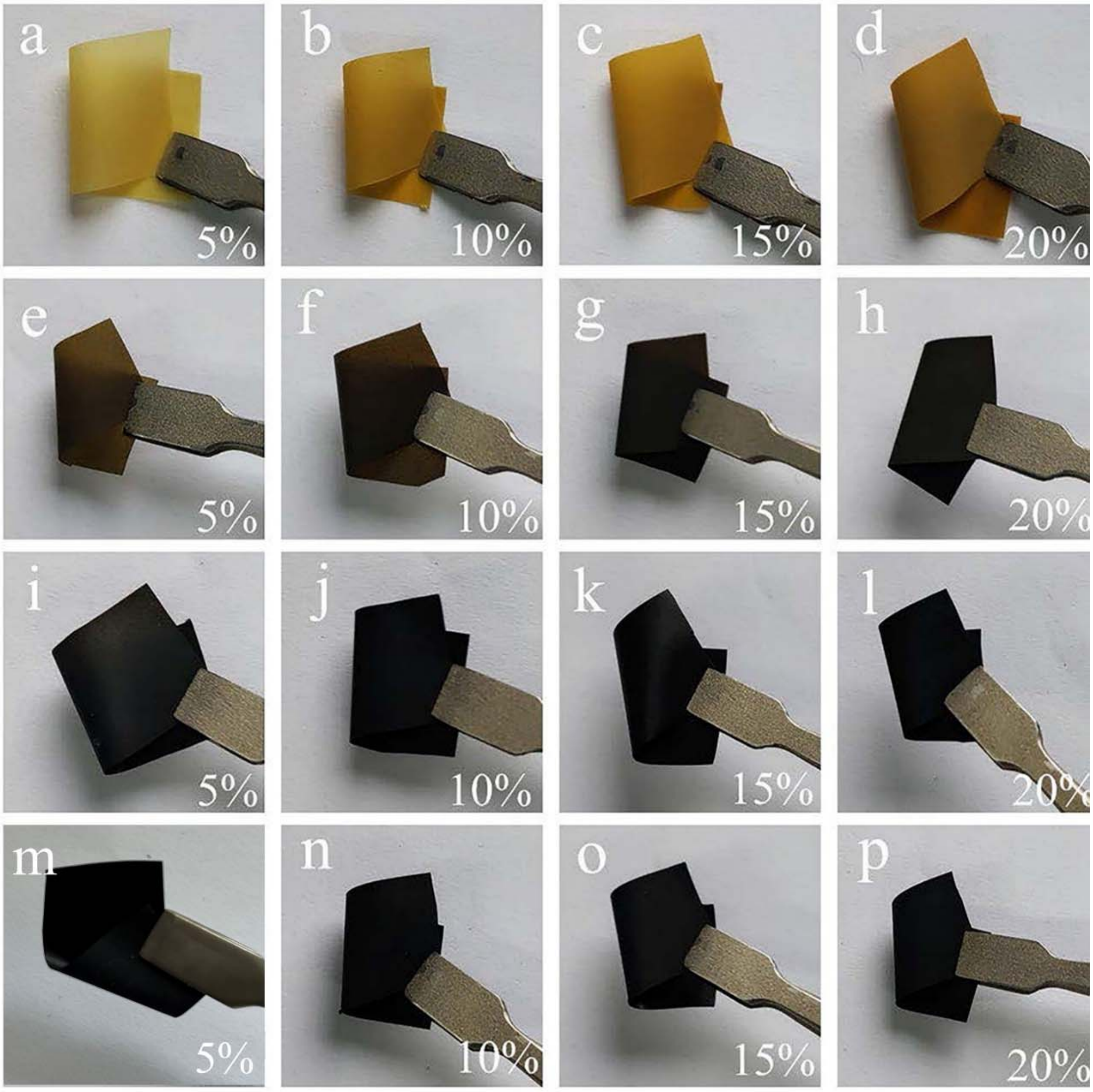

Figure S3. Pebax®1657-MOF-74(Ni) membranes with different filler loadings (**a**–**d**); Pebax®1657-MOF-74(Ni)@GO-1 MMMs with different filler loadings (**e**–**h**); Pebax®1657-MOF-74(Ni)@GO-2 MMMs with different filler loadings (**i**–**l**); Pebax®1657-MOF-74(Ni)@GO-3 MMMs with different filler loadings (**m**–**p**).

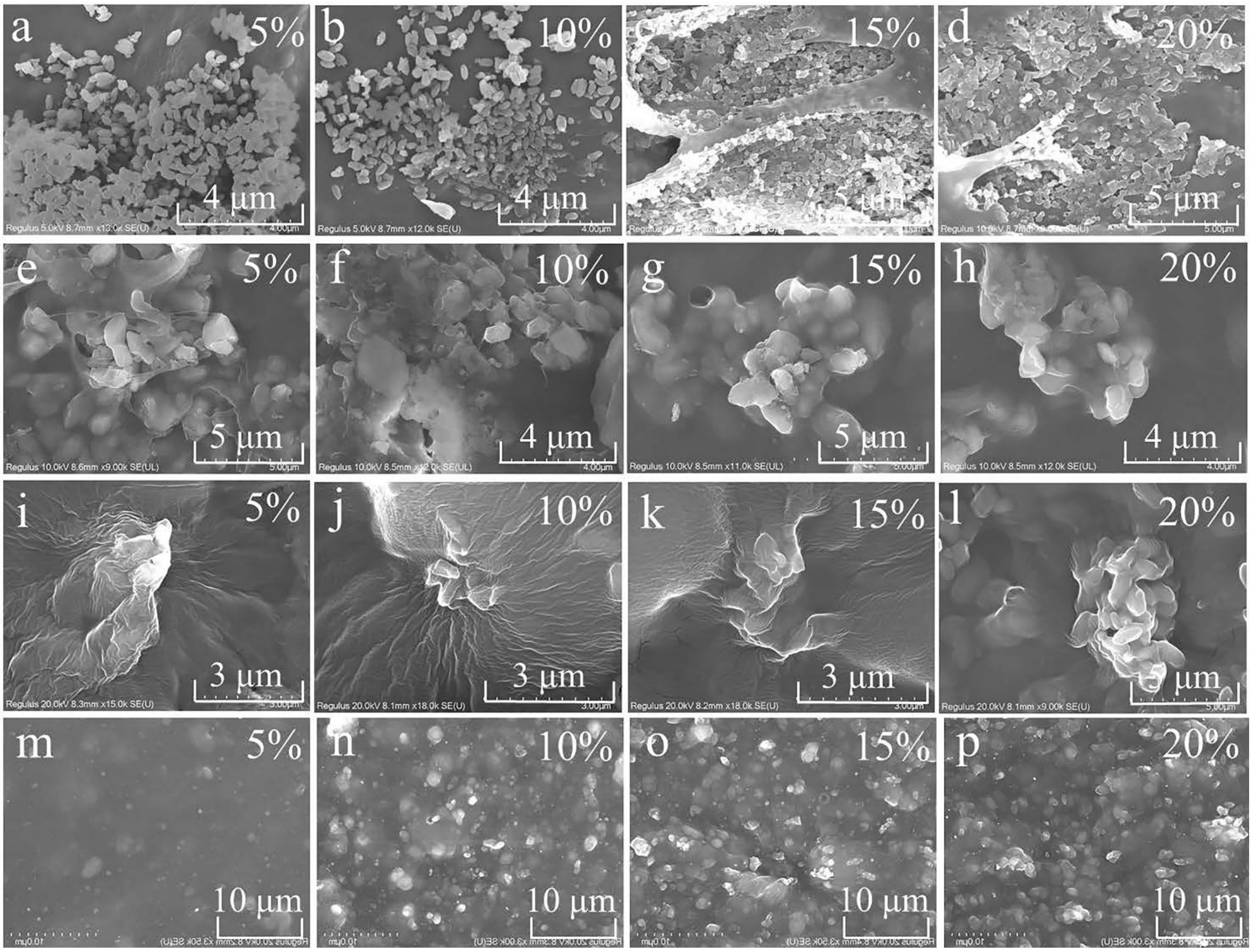

Figure S4. SEM images of Pebax®1657-MOF-74(Ni) membranes with different filler loadings (**a**–**d**); SEM images of Pebax®1657-MOF-74(Ni)@GO-1 MMMs with different filler loadings (**e**–**h**); SEM images of Pebax®1657-MOF-74(Ni)@GO-2 MMMs with different filler loadings (**i**–**l**); SEM images of Pebax®1657-MOF-74(Ni)@GO-3 MMMs with different filler loadings (**m**–**p**).

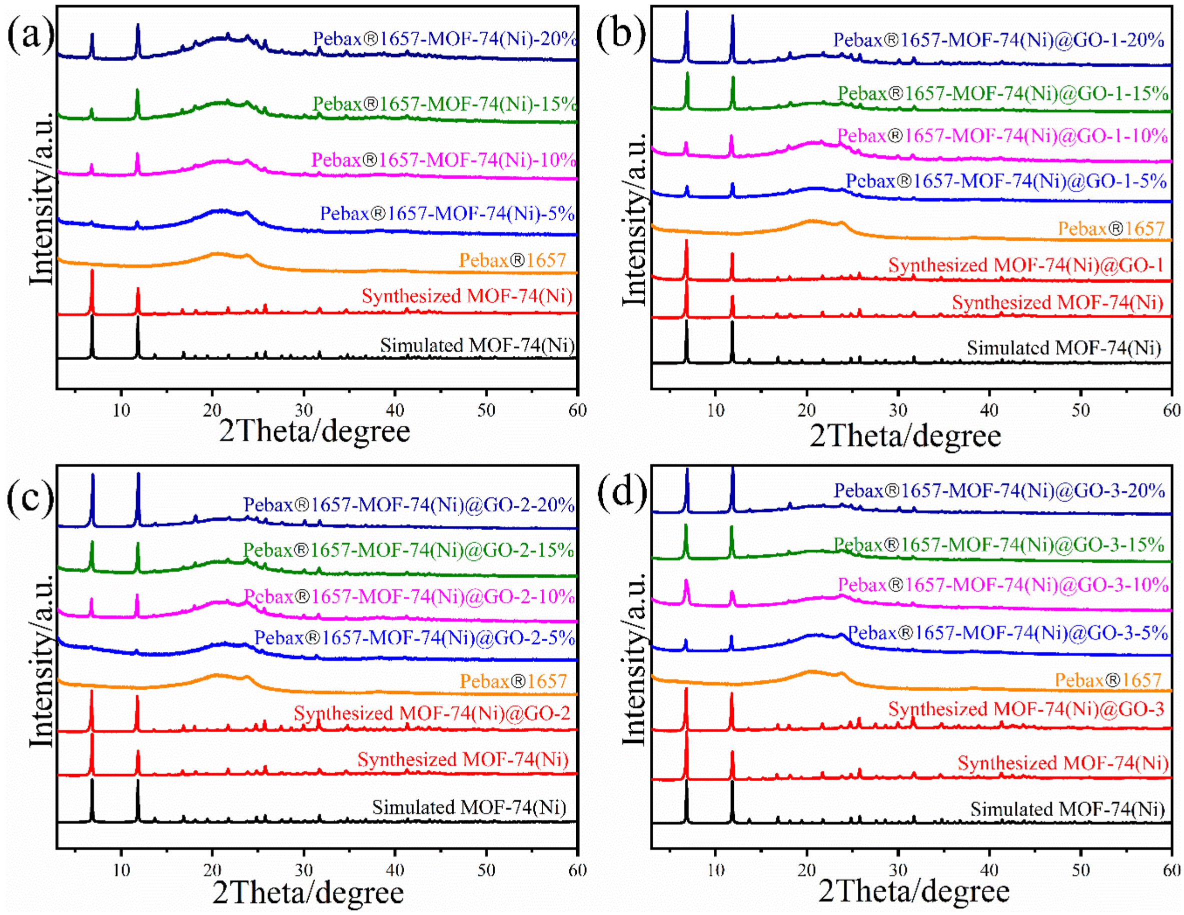

Figure S5. XRD patterns of composite membrane materials (**a**) MOF-74(Ni), (**b**) MOF-74(Ni)@GO-1, (**c**) MOF-74(Ni)@GO-2, and (**d**) MOF-74(Ni)@GO-3.

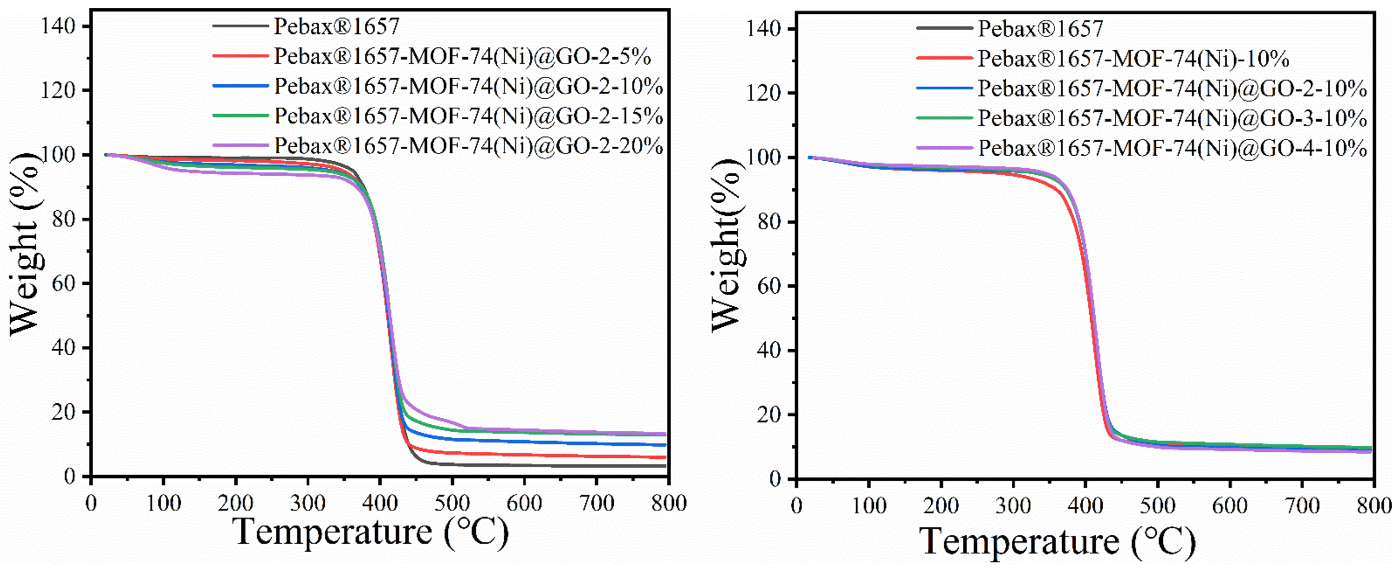

Figure S6. TGA curves of the membranes containing different (**a**) filler loading and (**b**) fillers.

Table S1. BET surface area and pore volume of MOF-74(Ni) and MOF-74(Ni)@GO with different GO loadings.

Samples	BET surface Area (m^2/g)	Micropore surface area (m^2/g)	Total pore volume (m^3/g)	Micropore volume (m^3/g)
MOF-74(Ni)	482.1	354.2	0.28	0.15
MOF-74(Ni)@GO-1	563.9	479.8	0.31	0.19
MOF-74(Ni)@GO-2	837.3	747.8	0.42	0.29
MOF-74(Ni)@GO-3	344.0	226.4	0.28	0.10

Table S2. The thickness of MMMs.

Membrane Sample	Load (wt%)	Thickness (μm)
Pebax®1657	--	74
Pebax®1657-MOF-74(Ni)	5	62
	10	88
	15	92
	20	98
Pebax®1657-MOF-74(Ni)@GO-1	5	86
	10	62
	15	72
	20	86
Pebax®1657-MOF-74(Ni)@GO-2	5	75
	10	78
	15	82
	20	78
Pebax®1657-MOF-74(Ni)@GO-3	5	78
	10	75
	15	84
	20	78

Table S3. The permeability and ideal selectivity of gases for Pebax®1657 and mixed matrix membranes.

Membrane Sample	Load (wt%)	P(CO_2) (Barrer)	P(N_2) (Barrer)	α(CO_2/N_2)
Pebax®1657	--	70.50	2.723	25.89
Pebax®1657-MOF-74(Ni)	5	111.3	1.797	61.83
	10	121.2	1.848	65.58
	15	125.1	1.866	67.04
	20	124.8	1.937	64.43
Pebax®1657-MOF-74(Ni)@GO-1	5	73.27	2.258	32.44
	10	85.23	1.985	42.94
	15	85.81	1.829	46.92
	20	91.11	1.525	59.09
Pebax®1657-MOF-74(Ni)@GO-2	5	90.37	1.426	63.27
	10	64.27	0.8351	76.96
	15	63.23	0.8853	71.58
	20	60.09	1.702	35.31
Pebax®1657-MOF-74(Ni)@GO-3	5	69.45	1.971	32.23
	10	56.36	1.960	28.76
	15	50.38	1.782	28.27
	20	49.96	1.769	28.24

Table S4. The permeability and ideal selectivity of gases for Pebax®1657-GO, which is the same as the GO loading in Pebax®1657-MOF-74(Ni)@GO-2.

Membrane Sample	Load (wt%)	$P(CO_2)$ (Barrer)	$P(N_2)$ (Barrer)	$\alpha(CO_2/N_2)$
Pebax®1657-GO-2	5	72.92	3.362	21.69
	10	66.84	2.664	25.09
	15	66.41	2.167	30.64
	20	66.47	2.157	29.89

Table S5. The permeability and ideal selectivity of gases for MMMs with 10 wt% filler loadings.

Sample	$S(CO_2)$	$S(N_2)$	$D(CO_2)$	$D(N_2)$	$\alpha(CO_2/N_2)$
Pebax®1657	7.45	0.24	9.468	11.47	25.89
Pebax®1657-MOF-74(Ni)	9.99	0.12	12.23	12.13	65.58
Pebax®1657-MOF-74(Ni) @GO-1	12.89	0.48	6.61	4.18	42.94
Pebax®1657-MOF-74(Ni) @GO-2	8.06	0.13	7.98	6.38	76.96
Pebax®1657-MOF-74(Ni) @GO-3	7.867	0.15	7.07	1.63	28.76

Table S6. Comparison of gas separation performance of this work with those of reported MMMs.

Materials	$P(CO_2)$ (Barrer)	$\alpha(CO_2/N_2)$	Testing conditions	Ref.
Peabx/ZIF-8@GO-6	249	47.6	1 bar, 25 °C	[47]
PSF-V-ZIF-8	89.7	30	3 bar, 30 °C	[48]
PSF-A@S/ZIF-8	4.25	27.78	4 bar, 30 °C	[49]
PSF-UiO-66-NH2@ZIF-8	48.2	25	3 bar, 35 °C	[50]
